# Surface-Induced ARGET ATRP for Silicon Nanoparticles with Fluorescent Polymer Brushes

**DOI:** 10.3390/polym11071228

**Published:** 2019-07-23

**Authors:** Chun-Na Yan, Lin Xu, Qing-Di Liu, Wei Zhang, Rui Jia, Cheng-Zhi Liu, Shuang-Shuang Wang, Li-Ping Wang, Guang Li

**Affiliations:** 1 College of Materials Science and Engineering, Liaocheng University, Liaocheng 252059, China; 2 College of Materials Science and Engineering, Qingdao University, Qingdao 266071, China

**Keywords:** dispersibility, rhodamine B, SI-ARGET ATRP, silica nanoparticles

## Abstract

Well-defined polymer brushes attached to nanoparticles offer an elegant opportunity for surface modification because of their excellent mechanical stability, functional versatility, high graft density as well as controllability of surface properties. This study aimed to prepare hybrid materials with good dispersion in different solvents, and to endow this material with certain fluorescence characteristics. Well-defined diblock copolymers poly (styrene)-*b*-poly (hydroxyethyl methyl acrylate)–*co*-poly (hydroxyethyl methyl acrylate- rhodamine B) grafted silica nanoparticles (SNPs-*g*-PS-*b*-PHEMA-*co*-PHEMA-RhB) hybrid materials were synthesized via surface-initiated activators regenerated by electron transfer atom transfer radical polymerization (SI-ARGET ATRP). The SNPs surfaces were modified by 3-aminopropyltriethoxysilane (KH-550) firstly, then the initiators 2-Bromoisobutyryl bromide (BIBB) was attached to SNPs surfaces through the esterification of acyl bromide groups and amidogen groups. The synthetic initiators (SNPs-Br) were further used for the SI-ARGET ATRP of styrene (St), hydroxyethyl methyl acrylate (HEMA) and hydroxyethyl methyl acrylate-rhodamine B (HEMA-RhB). The results indicated that the SI-ARGET ATRP initiator had been immobilized onto SNPs surfaces, the Br atom have located at the end of the main polymer chains, and the polymerization process possessed the characteristic of controlled/“living” polymerization. The SNPs-*g*-PS-*b*-PHEMA-*co*-PHEMA-RhB hybrid materials show good fluorescence performance and good dispersion in water and EtOH but aggregated in THF. This study demonstrates that the SI-ARGET ATRP provided a unique way to tune the polymer brushes structure on silica nanoparticles surface and further broaden the application of SI-ARGET ATRP.

## 1. Introduction

Polymer/silica nanoparticles (polymer/SNPs) hybrid materials combine the advantages of SNPs (thermal stability, rigidity, ease of modification) and polymers (ductility, functionality, machinability, compatibility) [1,2,3]. A variety of methods have been developed for preparation polymer/SNPs hybrid nanoparticles, including the in-situ introduction or adsorption of nanoparticles [4], the sol-gel technique [5,6], layer-by-layer deposition [7] and graft polymerization [8,9]. Among these methods, graft polymerization is a very popular potential method to form a film layer (perhaps polymers) that are covalently bonded onto the substrate surface [10,11,12], this modified surface shows good mechanical and chemical stability, as well as structural controllable. Within various methods of graft polymerization, surface-initiated atom transfer radical polymerization (SI-ATRP) [13,14,15,16] has been widely applied for grafting polymers on different materials surface, especially for grafting a densely anchored polymer shell with a high degree of control on the size, structure, and unique uniformity of the polymer chains, which was owing to the high compatibility of SI-ATRP process has a wide variety of monomers, molecular weight (MW) and structure designability, mild reaction conditions, well-defined thickness, and composition [17,18,19].

However, traditional ATRP is sensitive to oxygen and requires high transition-metal (Cu (I)) complex catalyst concentration to maintain activity throughout the polymerization process, which results in an inevitable metal residue in final products and is harmful to physiological systems [20]. Several new ATRP techniques were developed to solve these drawbacks in recent years, especially the technique of activators regenerated by electron transfer for ATRP (ARGET ATRP), can effectively decrease the amount of metal catalyst required by introducing excess reducing agent, the ARGET ATRP technique allows the utilization of ppm level of Cu catalysts to greatly reduce the sensitivity of oxygen, with good prospects for use in industry [21,22,23,24,25].

Silica nanoparticles (SNPs) due to their thermal stability and high rigidity as well as chemical resistance [26,27] and physical [28,29] have received much attentions. However, their poor hydrophobicity and dispersibility [30,31] have been largely restricted in many application fields. Therefore, the surface modification of SNPs with different polymers to improve their dispersibility in various solvents and extend their physicochemical properties is very essential. It has been proved by researchers’ continuous exploration that SNPs surface modified with organic materials to form silica/polymer core/shell nanohybrids [32,33,34], which also named polymer-modified SNPs (PM-SNPs), have been applied in various areas such as electrical materials and catalysis [35,36,37,38], optical performance [39,40], drug delivery [41,42], chain end fluorescein labeling [43,44] and so on.

Herein, we describe a synthesis of well-defined PS-*b*-PHEMA-*co*-PHEMA-RhB-grafted SNPs (SNPs-*g*-PS-*b*-PHEMA-*co*-PHEMA-RhB) via surface-initiated ARGET ATRP (SI-ARGET ATRP). Firstly, the ARGET ATRP initiator was grafted from the SNPs surface and used to initiate styrene polymerization on SNPs surface, which can make resulted in SNPs-g-PS hybrid materials. Owing to the livingness of the ATRP process [45,46,47] (which also can be verified by 1H-NMR in this work), the end group of the PS block can be reinitiated to continue the polymerization on SNPs surface with the hydrophilic monomer, HEMA and HEMA-RhB, resulting in the SNPs-*g*-PS-*b*-PHEMA-*co*-PHEMA-RhB. The purpose of this study is to obtain hybrid materials with good dispersion in different solvents, and to endow this material with certain fluorescence characteristics, so as to further broaden the application of SI-ARGET ATRP.

## 2. Materials and Methods

### 2.1. Materials

The styrene (St, AR) was obtained from Shanghai Chemical Reagent Plant (Shanghai, China) and distilled under reduced pressure before use. 2-Bromoisobutyryl bromide (BIBB), ethyl 2-bromo-2-methylpropionate (EBiB), rhodamine B (RhB) and triethylamine (TEA) was acquired from Shanghai Aladdin Chemical Reagent Co. Ltd. (Shanghai, China). 2-hydroxyethyl methacrylate (HEMA), *N,N,N′,N′,N*″-pentamethyl diethylenetriamine (PMDETA) were purchased from Sigma-Aldrich (Shanghai, China). ascorbic acid (Vc), tetrahydrofuran (THF), ethanol (EtOH) and other chemicals were purchased from Sinopharm Chemical Reagent Co., Ltd. (Shanghai, China). and used without further purification. All other chemical reagents were analytical grade and used without further purification.

### 2.2. Instrumental Characterization

Fourier Transform Infrared Spectrum (FT-IR) of the samples was achieved using FTIR Spectroscopy on a Nicolet iS5 (Thermo Fisher Scientific, Madison, WI, USA) spectroscopy, within a range of 500–4000 cm^−1^. X-ray photoelectron spectra (XPS) were taken on an Escalab 250xi spectrometer (Thermo Fisher Scientific, waltham, MA, USA) equipped with an Al Kα X-ray source (hν = 1486.6 eV). Proton nuclear magnetic resonance (^1^H NMR) spectra were recorded on a 400 MHz (Varian Mercury Plus 400, Palo Alto, CA, USA) nuclear magnetic resonance instrument, using CDCl_3_ and CD_3_OD as solvent and tetramethylsilane (TMS) as the internal reference. The molecular weights and molecular distributions of the polymers were measured at an alliance GPC 1515 (Waters 1515, Milford, MA, USA) gel permeation chromatographer (GPC) using THF as the eluent at a flow rate of 0.6 mL/min^−1^. Thermogravimetry analysis (TGA) of the materials was recorded on an STA 449C simultaneous DSC-TGA (Netzsch Instruments, Selb, Germany) with a heating rate of 10 °C min^−1^ in a nitrogen atmosphere and between the 30–800 °C temperature. The ultra-violet (UV-vis) images were taken on a Portable dark box UV analyzer (Jiapeng Instruments, Shanghai, China) at 365 nm.

### 2.3. Preparation of the Hydrophilic Monomer with Rhodamine B (HEMA-RhB)

**Figure 1:**
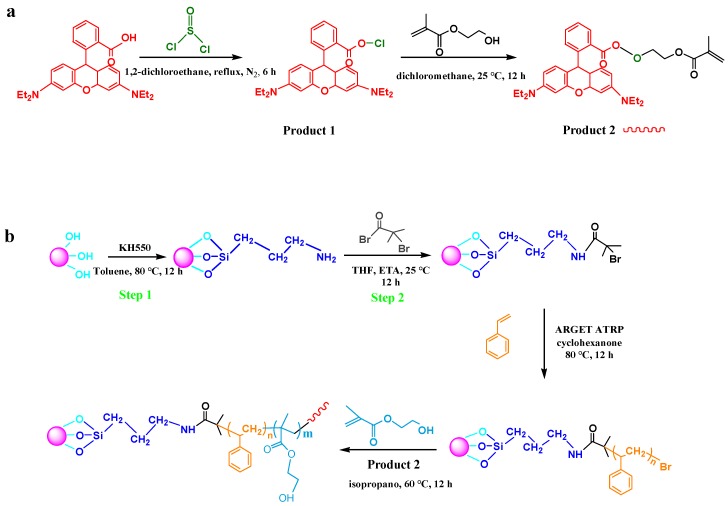
General synthetic procedure to fabricate SNPs-*g*-PS-*b*-PHEMA-RhB: (**a**) Synthesis of the HEMA-RhB (Product 2) and (**b**) Synthesis of SNPs-*g*-PS-*b*-PHEMA-RhB via surface-initiated.

The synthesis of monomer was prepared through two painstaking steps of the reaction. (The reaction process is shown in Figure 1a). First, rhodamine B was acylated chlorination by sulfoxide chloride. Rhodamine B 19.1 mg (0.03996 mmol) was dissolved in 4 mL 1, 2-dichloroethane, and sulfoxide chloride 30 μL (0.676 mmol) was added rapidly under nitrogen atmosphere. The reaction was heated to reflux and equipped with an absorption device for 6 h. After the reaction, the solvent and unreacted SOCl_2_ were decompressed to obtain the product 1 (RhB-Cl). Then, the resulting RhB-Cl and 2-Hydroxyethyl methacrylate (0.24 mL, 0.00198 mol) was dissolved in 3 mL dichloromethane, the reaction was treated for 12 h at room temperature. Finally, the resulted HEMA-RhB was concentrated and stored at room temperature in the dark.

### 2.4. Anchoring of the ARGET ATRP Initiator (Bromo-Initiator) on SNPs Surfaces

Silicon nanoparticles (SNPs) surface was modified with KH550 and 2-bromoisobutyryl bromide orderly. Following the typical steps: first, SPNs (1 g) was dispersed in 50 mL toluene, the KH550 was added into the dispersion liquid slowly. After treated at 80 °C for 12 h, the crude SNPs-KH550 was separated by centrifugation and washed completely by Soxhlet extraction with ethanol. The obtained SNPs-KH550 was dried at 40 °C under vacuum. XPS Calcd. (Atomic %): Si, 10.38; C, 18.61; N, 7.74; O, 62.97; Br, 0.3; N, 4.680 mmol g^−1^ (calculated according to the N atomic %) (Table 1).

Second, SNPs-KH550 0.7 g was dispersed in a round flask with 25 mL toluene containing 2 mL (0.014 mol) of TEA. Then 1.43 mL (0.0115 mol) of 2-bromoisobutyryl bromide (the mole ratio of TEA: BIBB with 1.5:1.2) was added dropwise into the reaction mixture at 0 °C keeping 2 h, then, the reaction was degassed at 25 °C for 12 h. Thereafter, the products were separated by centrifugation and the obtained solid (crude SNPs-Br) was rinsed with deionized water, ethanol thoroughly. The resulted SNPs-Br was dried at 40 °C in a vacuum. XPS Calcd. (Atomic %): Si, 8.93; C, 20; N, 7.18; O, 55.82; Br, 8.07; Br, 3.781 mmol g^−1^ (calculated according to the Br atomic %) (Table 1).

### 2.5. Synthesis of Silica-Grafting-Polystyrene (SNPs-g-PS)

Figure 1b shows the general synthetic routine to fabricate polystyrene-grafted SNPs via SI-ARGET ATRP, SNPs-*g*-PS. In brief, SNPs-Br (0.15 g, 0.567 mmol Br groups) dispersed into 5 mL cyclohexanone. Then, styrene (4 mL, 0.035 mol), PMDETA (14.5 µL 0.0698 mmol), EBiB (10 µL, 0.0698 mmol), catalytic agent (CuCl_2_·2H_2_O) (1.2 mg, 0.007 mmol) was added in these reactions with the mole ratio with 500:1:1:0.1, after which the flask system was deoxygenated by degassing and backfilling with argon and stirring under ambient conditions up to homogeneity. After that, the reaction treated at 90 °C in an oil bath for 12 h. The products were collected by centrifugation, obtained two parts with a clear liquid (which contains the polystyrene homopolymer in solution) and sediment (which was named crude SNPs*-g*-PS). The clear liquid was precipitated in methanol to obtain polystyrene homopolymer. On the other hand, sediment (SNPs-*g*-PS) was washed with THF via Soxhlet extraction for 24 h to remove the ungrafted PS completely and dried at 40 °C under vacuum.

### 2.6. Preparation of the Fluorescence SNPs-g-PS-b-PHEMA-co-PHEMA-RHB Via SI-ARGET ATRP

Typical batch of SI-ARGET ATRP polymerization was charged with the molar ratio of 300:1:0.1:1:0.05 for [M]: [EBiB]: [CuCl_2_·2H_2_O]: [PMDETA]: [Vc]. In round bottom flak, SNPs-*g*-PS (72.6 mg) was well dispersed in the isopropanol (2 mL) and then the 2-Hydroxyethyl methacrylate (4.6 mL, 0.038 mol), HEMA-RhB, CuCl_2_·2H_2_O (2.2 mg, 0.0126 mmol) and PMDETA (26.4 μL, 0.126 mmol) were added into the suspension orderly. The mix was degassed and back-filled with nitrogen, subsequently, the solutions of reducing agent Vc (1.1 mg, 0.0063 mmol) and initiator EBiB (18.5 μL, 0.126 mmol) were injected into the flask. The reaction bottle was placed into an oil bath at 60 °C for 8 h. After the reaction, the system was opened into the air and separated by centrifugation. The obtained solid (crude SNPs-g-PS-*b*-PHEMA-*co*-PHEMA-RHB) was precipitated in cold diethyl ether and extracted with ethanol to remove the ungrafted polymers completely and dried at 40 °C in vacuum. The solution after centrifugation was precipitated in cold diethyl ether and dried at 30 °C in a vacuum, resulting in PHEMA-*co*-PHEMA-RHB. The detailed reaction process was listed in Figure 1.

## 3. Results and Discussion

### 3.1. Structure Analysis

**Figure 2:**
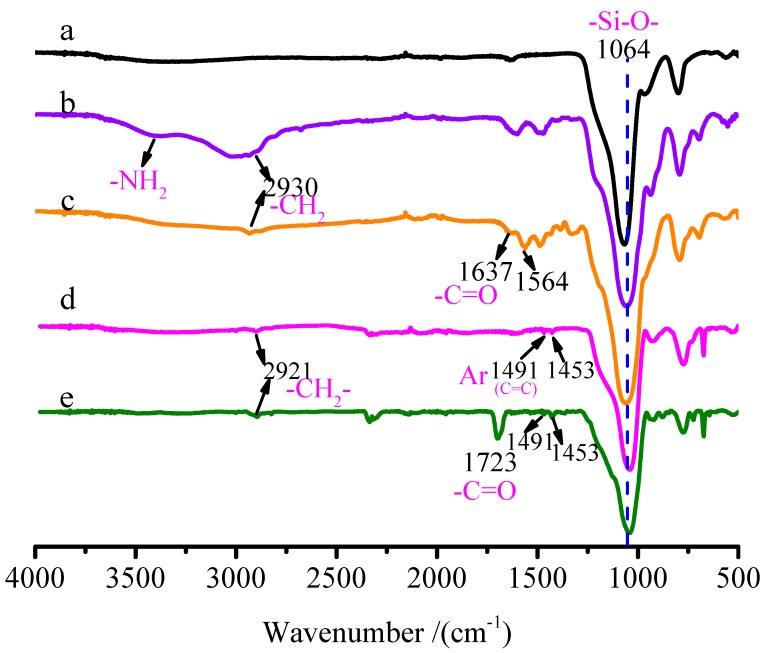
FT-IR spectrum of (**a**) SNPs, (**b**) SNPs-KH550, (**c**) SNPs-Br, (**d**) SNPs-*g*-PS, (**e**) SNPs-*g*-PS-*b*-PHEMA-*co*-PHEMA-RhB.

The hydroxyl groups on SNPs surface can hydrolyze with the activation groups –OC_2_H_5_ of KH550, then the products contain -NH_2_ on SNPs surface, which is advantageous for ATRP initiator BiBB immobilized to the SNPs surface. Figure 2 shows the FT-IR spectrum of (a) pure SNPs, (b) SNPs-KH550, (c) SNPs-Br, (d) SNPs-*g*-PS and (e) SNPs-*g*-PS-*b*-PHEMA-*co*-PHEMA-RhB. The bands at 1064 cm^−1^ are attributed to the stretching vibration absorption of Si–O–Si in SNPs. In Figure 2b, the bands emerged at 3400 cm^−1^ are owing to the –NH_2_, the bands at 2930 cm^−1^ are corresponding to the C–H stretching vibrations. These results demonstrate the surfaces of SNPs were modified with silane coupling agent KH550. Compared with Figure 2b, the new emerged bands at 1637 cm^−1^ is attributed to the NH–C=O stretching vibration of BiBB on SNPs surface, indicating the successful immobilization of BiBB to SNPs surface (Figure 2c). Figure 2d shows the FT-IR spectrum of SNPs-*g*-PS: the bands at 1453 and 1491cm^−1^ are attributed to C=C stretching vibrations of the benzene ring in PS, which confirm that polystyrene has been grafted onto SNPs surface. As for the diblock copolymer-grafted SNPs: SNPs-*g*-PS-*b*-PHEMA-*co*-PHEMA-RhB (Figure 2e), the C=C stretching vibrations in PS are still there. The new bands at 1723 cm^−1^ belongs to ester carbonyl C=O stretching vibration of PHEMA in SNPs-*g*-PS-*b*-PHEMA-*co*-PHEMA-RhB, which indicated that PHEMA been grafted from SNPs surface.

**Figure 3:**
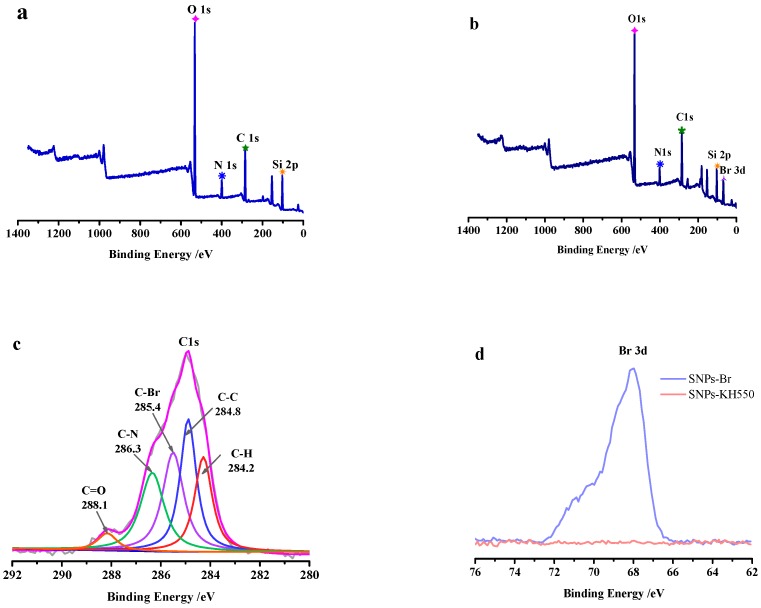
XPS spectra of SNPs-KH550 (**a**), and SNPs-Br (**b**–**d**): Survey scan curves range from 0 to 1400 eV (**b**), the C1s core-level spectra (**c**) and Br3d region (**d**).

Figure 3a,b show the full spectrum of XPS before (SNPs-KH550) and after (SNPs-Br) modification respectively. It can be seen from the spectrum that they mainly contain Si, C, O and N. In addition, there is an obvious difference between them: the signal of Br 3d appears at 68 eV in Figure 3b,d. Expect that the binding type of C 1s also changed as shown in Figure 3c, the C 1s of SNPs-Br consisted with five types bond, which contains C–C, C–H, C–N, C=O and C-Br, accompanied by the appearance of peak at 284.8, 284.2, 286.3 and 285.4 eV, respectively. The existence of C=O and C–Br indicated that the -NH_2_ groups on the surface have reacted with the acyl bromide groups in BiBB to form amide bonds, which corroborates with the peaks (–C=O) in Figure 2c. Furthermore, the element compositions (atomic %) of SNPs surface based on XPS obtain were listed in Table 1. From Table 1, it is easy to see the changes of the elements intuitively, especially bromine, which provides a guarantee for the following SI-ARGET ATRP.

**Figure 4:**
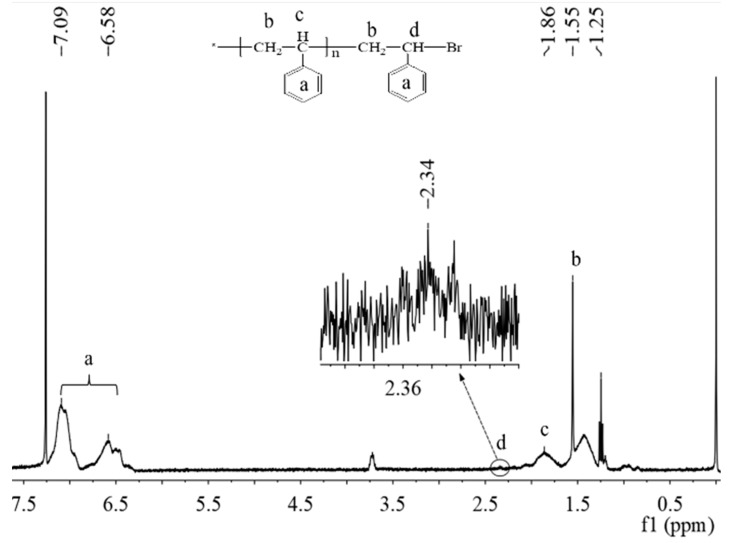
^1^H NMR spectrum of PS formed in solution.

**Figure 5:**
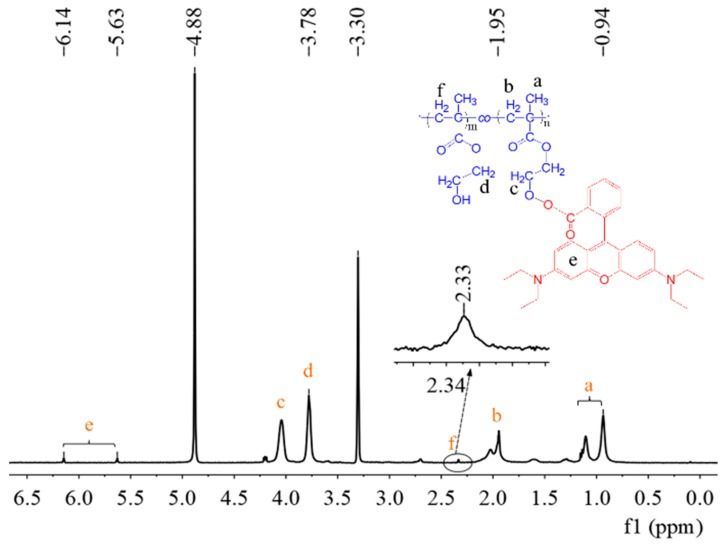
^1^H NMR spectrum of PHEMA-*co*-PHEMA-RhB formed in solution.

The monomer polymerized was obtained simultaneously by sacrificing the initiator in the solution. This has some theoretical support for us to understand the reaction process. Figure 4 and Figure 5 show the ^1^H NMR spectrum of PS and PHEMA-*co*-PHEMA-RhB. It is worth noting that the solvent is different between PS and PHEMA-*co*-PHEMA-RhB, the CDCl_3_ and CD_3_OD were used respectively. In general, the peak of NMR internal reference (TMS) at 0.00 ppm and CDCl_3_ at 7.27 ppm (Figure 4), CD_3_OD at 3.30 ppm (Figure 5). In Figure 4, except for the signals at around 1.85 ppm and 1.25–1.55 ppm are ascribed to the protons of methylene and methyl to the repeat unit in the main polystyrene chain. The peak at 6.58 and 7.09 ppm are attributed to the hydrogen on the benzene. Simultaneously, a weak signal at 2.36 ppm was also detected, which is the contribution of hydrogen to secondary carbon at the end of the main chain. As expected, due to the presence of Br atom, the density of the electron cloud near the Br decreases, which results in the resonance signal moving to the low field at 2.34 ppm. In addition, compared with the peak at 1.95 ppm form the –CH_2_– of PHEMA-*co*-PHEMA-RhB main chain, the peak at 2.34 ppm was also due to the Br influence (Figure 5). This proved the mechanism of SI-ARGET ATRP furthermore. Table S1 shows the molecular weight and distribution of PS formed in solution. The molecular weight (Mn) values reaches 6995 g mol^−1^ and the molecular weight distribution (M_w_/M_n_) is narrow (1.20), which indicated that the polymerizations possess the characteristic of controlled polymerization.

### 3.2. Morphology Analysis

**Figure 6:**
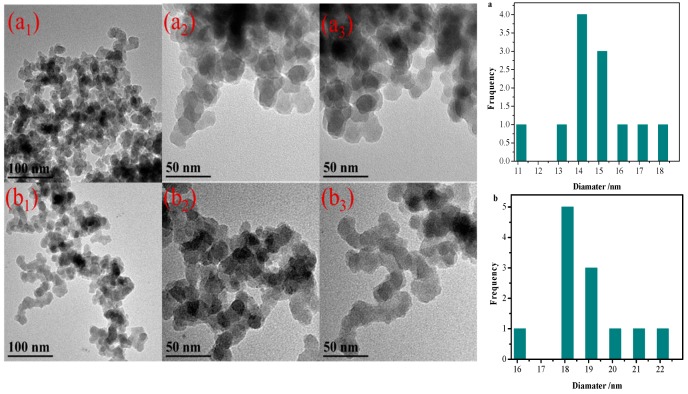
TEM images of (**a_1_**–**a_3_**) SNPs, (**b_1_**–**b_3_**) SNPs-*g*-PS and the particle size distribution.

In order to intuitively observe the changes of SNPs particle size and distribution morphology before (SNPs) and after grafting (SNPs-*g*-PS), the changes of particle size distribution were analyzed by TEM and Nano Measurer software. Figure 6 shows the TEM images of SNPs and SNPs-*g*-PS. It can be observed that the diameter tends to increases with the process of grafting polymerization, as shown in Figure 6a,b. The average diameter of the pure SNPs mainly centered at 14–15 nm. The smaller size has higher specific surface energy, which makes them easy to agglomerate and less to dispersible as shown in Figure 6a_1_–a_3_. This phenomenon hinders the application of SNPs. But after grafting PS by SI-ARGET ATRP, as shown in Figure 6b_1_–b_3_, the SNPs-*g*-PS diameter increased, mainly distribution at 18–19 nm, and the degree of dispersion is better than before. This is due to that the hydrophobic groups of polystyrene grafted on SNPs surface allow them to interact with each other to facilitate dispersion. Regardless of particle size or dispersion, the introduction of the polymer increases the functionality of SNPs, which indirectly indicates that the grafting of SNPs on the surface by SI-ARGET ATRP was successful.

### 3.3. TGA Analysis

**Figure 7:**
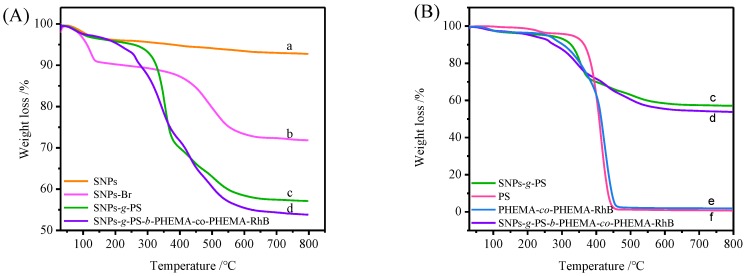
(**A**) TGA curves of (a) SNPs, (b) SNPs-Br, (c) SNPs -*g*-PS, (d) SNPs-*g*-PS-*b*-PHMEA-*co*-PHMEA-RhB. (**B**) TGA curves of (c) SNPs -*g*-PS, (d) SNPs-*g*-PS-*b*-PHMEA-*co*-PHMEA-RhB, (e) PHEMA-*co*-PHEMA-RhB and (f) PS.

The TGA was employed to investigate the thermostability of the hybrid materials and the grafting percentage of polymers on SNPs surface. Figure 7 shows the TGA of the SNPs, SNPs-Br, SNPs-*g*-PS, SNPs-*g*-PS-*b*-PHMEA-*co*-PHMEA-RhB, pure polymers PS and PHEMA-RhB with temperature increase from 30 to 800 °C. The first weight loss below 200 °C is due to the solvent evaporation in the samples. The significant weight loss stage appeared at the range of 200–700 °C is associated with the thermal decomposition of the organic content in the samples (the thermal decomposition temperature of pure PS and pure PHMEA-*co*-PHMEA-*co*-PHMEA-RhB is 450 °C, above 450 °C all the organic residues will decompose, with the breakage of organosilica bonds). The pure SNPs show no obvious decomposition (7.0%) between 30–800 °C. Therefore, the grafting percentage of polymers on the SNPs surfaces can be obtained via the weight loss of 200–700 °C. The organic content calculated for SNPs-Br, SNPs-*g*-PS and SNPs-*g*-PS-*b*-PHMEA-*co*-PHMEA-RhB is 17.73%, 38.47% and 41.47% respectively. That is, the grafted PS and the grafted PHMEA-*co*-PHMEA-RhB on SNPs surface is about 20.74% and 3.01% respectively. These results indicated that polymers have grafted from SNPs surface by SI-ARGET ATRP successfully.

### 3.4. Fluorescence and Dispersibility Analysis

**Figure 8:**
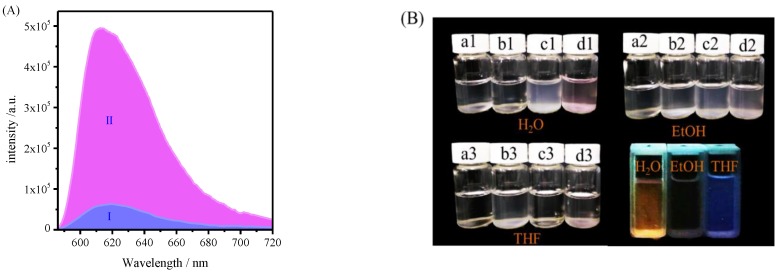
(**A**) The fluorescence spectrum of Ⅰ SNPs-*g*-PS-*b*-PHMEA-*co*-PHMEA-RhB, Ⅱ PHMEA-*co*-PHMEA-RhB formed in solution. (**B**) The dispersibility of SNPs (a1, a2, a3), SNPs-Br (b1, b2, b3), SNPs-*g*-PS (c1, c2, c3), SNPs-*g*-PS-*b*-PHMEA-*co*-PHMEA-RhB (d1, d2, d3) with concentration 0.33 mg/mL in H_2_O, EtOH, THF solutions, respectively, and the UV-irradiation picture of SNPs-*g*-PS-*b*-PHMEA-*co*-PHMEA-RhB in H_2_O(1), EtOH(2), THF(3) three difference solutions at 365 nm.

Figure 8 presents the fluorescence of SNPs-*g*-PS-*b*-PHMEA-*co*-PHMEA-RhB and PHMEA-*co*-PHMEA-RhB formed in solution, and the images of the dispersions of SNPs (a1, a2, a3), SNPs-Br (b1, b2, b3), SNPs-*g*-PS (c1, c2, c3), SNPs-*g*-PS-*b*-PHMEA-co-PHMEA-RhB (d1, d2, d3) in H_2_O, EtOH, THF solutions, and the UV-irradiation of SNPs-*g*-PS-*b*-PHMEA-*co*-PHMEA-RhB in three difference solutions. From Figure 8A, it can be observed obviously thatⅠhas a range of fluorescence emission peak at about 618 nm, which is in accord with the RhB characteristic emission peak as well as Ⅱ. The pure SNPs dispersed in water and EtOH, but the mass of shrinkage in THF. The grafting of PS onto SNPs surfaces (c1, c2, c3) resulted in completely opposite dispersibility. After grafting the PHEMA-RhB from SNPs surfaces, good dispersion in water and EtOH but aggregated in THF appeared, which was owing to the contribution of the amphiphilic PHEMA-co-PHEMA-RhB chains. SNPs-*g*-PS-*b*-PHMEA-*co*-PHMEA-RhB appearance jacinth and royal purple in H_2_O, THF respectively at 365 nm UV-irradiation light, but quenching in EtOH. These all instructions that the monomer with RhB successfully grafted. 

## 4. Conclusions

The controllable growth of polymer from SNPs surface was realized by “grafting from” method. Firstly, the initiator 2-bromoisobutyryl bromide was immobilized on the surface of SNPs, and the controlled of polystyrene was grafted on SNPs surface by surface-induced ARGET ATRP. The chain end functionality of SNPs-*g*-PS was confirmed and was used as a macroinitiator for further preparation of SNPs-*g*-PS-*b*-PHEMA-*co*-PHEMA-RhB hybrid materials. The PDI was narrow which indicated that the polymerization process possesses the characteristic of controlled/“living” polymerization. In addition, the grafted SNPs were well dispersed in water with a certain optical property, due to the presence of the PHEMA hydrophilic group and the rhodamine B fluorescent group. This work provides an effective strategy for solving the dispersion of small-sized nanoparticles by grafting a polymer brush, and also realizes the design and preparation of multi-functionality hybrid material by the surface-induced ARGET ATRP method.

## Figures and Tables

**Table 1:** The element compositions (atomic %) of SNPs-KH550 and SNPs-Br from XPS.

	Elements	Si	C	N	O	Br
Sample						
SNPs-KH550		10.38	18.61	7.74	62.97	0.3
SNPs-Br		8.93	20	7.18	55.82	8.07

## Supplementary Materials

The following are available online at https://www.mdpi.com/2073-4360/11/7/1228/s1, Table S1: Molecular weight and distribution of PS in solution.

## References

[1] Sikora J.W., Gajdoš I., Puszka A. (2019). Polyethylene-Matrix Composites with Halloysite Nanotubes with Enhanced Physical/Termal Properties. Polymers.

[2] Yuan Y., Chen W., Ma Z., Deng Y., Chen Y., Chen Y., Hu W. (2019). Enhanced optomechanical properties of mechanochemiluminescent poly (methyl acrylate) composites with granulated fluorescent conjugated microporous polymer fillers. Chem. Sci..

[3] Jin J., Du X., Yu J., Qin S., He M., Zhang K., Yang J. (2019). Synthesis of Negatively Charged Polyol-Functional PSF Membranes with Good Hydrophilic and Efficient Boron Removal Properties. Polymers.

[4] Wang X., Song J., Zhao J., Wang Z., Wang X. (2019). In-situ active formation of carbides coated with NPTiO_2_ nanoparticles for efficient adsorption-photocatalytic inactivation of harmful algae in eutrophic water. Chemosphere.

[5] Shchipunov Y.A., Karpenko T.Y. (2004). Hybrid polysaccharide−silica nanocomposites prepared by the sol−gel technique. Langmuir.

[6] Lien S.Y., Wuu D.S., Yeh W.C., Liu J.C. (2006). Tri-layer antireflection coatings (SiO_2_/SiO_2_–TiO_2_/TiO_2_) for silicon solar cells using a sol–gel technique. Sol. Energy Mater. Sol. Cells.

[7] Xu W., Wang Z., Shi L., Ma Y., Yuan S., Sun L., Zhao Y., Zhang M., Zhu J. (2015). Layer-by-layer deposition of organic–inorganic hybrid multilayer on microporous polyethylene separator to enhance the electrochemical performance of lithium-ion battery. ACS Appl. Mater. Interfaces.

[8] Wu W., Niu H., Yang D., Wang S., Jiang N., Wang J., Hu C. (2018). Polyaniline/Carbon Nanotubes composite modified anode via graft polymerization and self-assembling for microbial fuel cells. Polymers.

[9] Wang Y., Li R., Jia H., Yang G., Di Y., Xu C., Ma L., Zhang H., Zhou Y., Zang Y. (2018). A novel silane coupling agent with peroxy groups used as an initiator in the graft polymerization of AN or MMA on nano-TiO_2_. Chem. Pap..

[10] Salmi-Mani H., Terreros G., Barroca-Aubry N., Aymes-Chodur C., Regeard C., Roger P. (2018). Poly (ethylene terephthalate) films modified by UV-induced surface graft polymerization of vanillin derived monomer for antibacterial activity. Eur. Polym. J..

[11] Schulz A.S., Gojzewski H., Huskens J., Vos W.L., Julius V.G. (2018). Controlled sub-10-nanometer poly (N-isopropyl-acrylamide) layers grafted from silicon by atom transfer radical polymerization. Polym. Adv. Technol..

[12] Liu Y., Dong M., Wang T., Xiong L., Hang T., Ling H., Li M. (2019). Design of thermally stable insulation film by radical grafting poly (methylacrylic acid) on silicon surface. Appl. Surf. Sci..

[13] Liu Y., Gu H., Jia Y., Liu J., Zhang H., Wang R., Zhang B., Zhang H., Zhang Q. (2019). Design and preparation of biomimetic polydimethylsiloxane (PDMS) films with superhydrophobic, self-healing and drag reduction properties via replication of shark skin and SI-ATRP. Chem. Eng. J..

[14] Yan C.N., Liu Q., Xu L., Bai L.P., Wang L.P., Li G. (2019). Photoinduced Metal-Free Surface Initiated ATRP from Hollow Spheres Surface. Polymers.

[15] Fan D., Wang W., Chen H., Bai L., Yang H., Wei D., Yang L., Xue Z., Niu Y. (2019). Self-healing and tough GO-supported hydrogels prepared via surface-initiated ATRP and photocatalytic modification. New J. Chem..

[16] He S., Wang H., Zhang C., Zhang S., Yu Y., Lee Y., Li T. (2019). A generalizable method for the construction of MOF@ polymer functional composites through surface-initiated atom transfer radical polymerization. Chem. Sci..

[17] Barbey R., Lavanant L., Paripovic D., Schuwer N., Sugnaux C., Tugulu S., Klok H.A. (2009). Polymer brushes via surface-initiated controlled radical polymerization: Synthesis, characterization, properties, and applications. Chem. Rev..

[18] Edmondson S., Osborne V.L., Huck W.T. (2004). Polymer brushes via surface-initiated polymerizations. Chem. Soc. Rev..

[19] Matyjaszewski K. (2012). Atom transfer radical polymerization (ATRP): Current status and future perspectives. Macromolecules.

[20] Gao H., Matyjaszewski K. (2007). Synthesis of molecular brushes by “grafting onto” method: Combination of ATRP and click reactions. J. Am. Chem. Soc..

[21] Simakova A., Averick S.E., Konkolewicz D., Matyjaszewski K. (2012). Aqueous arget atrp. Macromolecules.

[22] Wang S., Song J., Li Y., Zhao X., Chen L., Li G., Wang L., Jia Z., Ge X. (2019). Grafting antibacterial polymer brushes from titanium surface via polydopamine chemistry and activators regenerated by electron transfer ATRP. React. Funct. Polym..

[23] Guo M., Wu Y., Xue S., Xia Y., Zhang R., Liu D., Zhang T. (2019). Surface modification of boron nitride nanosheets with polycationic electrolytes through ARGET ATRP for enhancing mechanical properties of cellulose film. Mater. Lett..

[24] Dong H., Matyjaszewski K. (2008). ARGET ATRP of 2-(dimethylamino) ethyl methacrylate as an intrinsic reducing agent. Macromolecules.

[25] Chen M., Zhou H., Zhou L., Zhang F. (2017). Confined polymerization: ARGET ATRP of MMA in the nanoporesof modified SBA-15. Polymer.

[26] Ghasabkolaei N., Janalizadeh A., Jahanshahi M., Roshan N., Ghasemi S.E. (2016). Physical and geotechnical properties of cement-treated clayey soil using silica nanoparticles: An experimental study. Eur. Phys. J. Plus.

[27] Wu K.C.W., Yamauchi Y. (2012). Controlling physical features of mesoporous silica nanoparticles (MSNs) for emerging applications. J. Mater. Chem..

[28] Dwivedi S., Sakamoto S., Kato S., Mitsumata T., Kaneko T. (2018). Effects of biopolyimide molecular design on their silica hybrids thermo-mechanical, optical and electrical properties. RSC Adv..

[29] Ghiyasi S., Sari M.G., Shabanian M., Hajibeygi M., Zarrintaj P., Rallini M., Torre L., Puglia D., Vahabi H., Jouyandeh M. (2018). Hyperbranched poly (ethyleneimine) physically attached to silica nanoparticles to facilitate curing of epoxy nanocomposite coatings. Prog. Org. Coat..

[30] Chen B.K., Chiu T.M., Tsay S.Y. (2004). Synthesis and characterization of polyimide/silica hybrid nanocomposites. J. Appl. Polym. Sci..

[31] Hwang Y., Lee J.K., Lee J.K., Jeong Y.M., Cheong S.I., Ahn Y.C., Kim S.H. (2008). Production and dispersion stability of nanoparticles in nanofluids. Powder Technol..

[32] Cai Y., Peng W., Demeshko S., Tian J., Vana P. (2018). Silica-Coated Magnetite Nanoparticles Carrying a High-Density Polymer Brush Shell of Hydrophilic Polymer. Macromol. Rapid Commun..

[33] Tan L.L., Shang L. (2019). Smart Delivery Systems Based on Poly (glycidyl methacrylate) s-Coated Organic/Inorganic Core–Shell Nanohybrids. Macromol. Rapid Commun..

[34] Wei Q., Ji J., Shen J. (2008). Synthesis of near-infrared responsive gold nanorod/pnipaam core/shell nanohybrids via surface initiated atrp for smart drug delivery. Macromol. Rapid Commun..

[35] Dai J., Dong Y., Yu C., Liu Y., Teng X. (2018). A novel Nafion-g-PSBMA membrane prepared by grafting zwitterionic SBMA onto Nafion via SI-ATRP for vanadium redox flow battery application. J. Membr. Sci..

[36] Guo Q., Han Y., Wang H., Sun W., Jiang H., Zhu Y., Xie K. (2018). Thermo and electrochemical-stable composite gel polymer electrolytes derived from core-shell silica nanoparticles and ionic liquid for rechargeable lithium metal batteries. Electrochim. Acta.

[37] Farooqi Z.H., Ijaz A., Begum R., Naseem K., Usman M., Ajmal M., Saeed U. (2018). Synthesis and characterization of inorganic–organic polymer microgels for catalytic reduction of 4-nitroaniline in aqueous medium. Polym. Compos..

[38] Jamshidi A., Maleki B., Zonoz F.M., Tayebee R. (2018). HPA-dendrimer functionalized magnetic nanoparticles (Fe_3_O_4_@ D-NH_2_-HPA) as a novel inorganic-organic hybrid and recyclable catalyst for the one-pot synthesis of highly substituted pyran derivatives. Mater. Chem. Phys..

[39] Zhao S., Tao Y., Chen Y., Zhou Y., Li R., Xie L., Jin P., Ji S. (2019). Room-temperature Synthesis of Inorganic-organic Hybrid Coated VO_2_ nanoparticles for Enhanced Durability and Flexible Temperature-responsive Near-infrared Modulator Application. ACS Appl. Mater. Interfaces.

[40] Kang J., Kim D., Wang J., Han Y., Zuidema J.M., Hariri A., Park J.-H., Jokerst J.V., Sailor M.J. (2018). Enhanced performance of a molecular photoacoustic imaging agent by encapsulation in mesoporous silicon nanoparticles. Adv. Mater..

[41] Wilczewska A.Z., Niemirowicz K., Markiewicz K.H., Car H. (2012). Nanoparticles as drug delivery systems. Pharmacol. Rep..

[42] Bagheri E., Ansari L., Abnous K., Taghdisi S.M., Charbgoo F., Ramezani M., Alibolandi M. (2018). Silica based hybrid materials for drug delivery and bioimaging. J. Control. Release.

[43] Tas S., Kopeć M., van der Pol R., Cirelli M., de Vries I., Bölükbas D.A., Tempelman K., Benes N.E., Hempenius M.A., Vancso G.J. (2019). Chain End-Functionalized Polymer Brushes with Switchable Fluorescence Response. Macromol. Chem. Phys..

[44] Kopeć M., Tas S., Cirelli M., van der Pol R., de Vries I., Vancso G.J., de Beer S. (2019). Fluorescent Patterns by Selective Grafting of a Telechelic Polymer. ACS Appl. Polym. Mater..

[45] Herberg A., Yu X., Kuckling D. (2019). End Group Stability of Atom Transfer Radical Polymerization (ATRP)-Synthesized Poly(N-isopropylacrylamide): Perspectives for Diblock Copolymer Synthesis. Polymers.

[46] Wang S., Meng H., Li Y., Sun D., Zhan Y., Ge X., Chen L. (2019). Polymer brushes grafted from graphene via bioinspired polydopamine chemistry and activators regenerated by electron transfer atom transfer radical polymerization. J. Polym. Sci. Part A Polym. Chem..

[47] Kaßel M., Gerke J., Ley A., Vana P. (2018). Surface Modification of Wood Flour via ARGET ATRP and Its Application as Filler in Thermoplastics. Polymers.

