# Supplementary material for: Surface-Induced ARGET ATRP for Silicon Nanoparticles with Fluorescent Polymer Brushes
Source: Polymers (Basel). 2019 Jul 23;11(7):1228. doi: 10.3390/polym11071228 (PMC6680766; doi:10.3390/polym11071228)
Supplement: Supplementary file 1 [file polymers-11-01228-s001.pdf]

polymers

Article- Supporting information

Surface-Induced ARGET ATRP for Silicon Nanoparticles with Fluorescent Polymer Brushes

Chun-Na Yan[1], Lin Xu[2], Qing-Di Liu[1], Wei Zhang[1], Rui Jia[1], Cheng-Zhi Liu[1], Shuang-Shuang Wang[1], Li-Ping Wang[1,*] and Guang Li[1,*]

[1] College of Materials Science and Engineering, Liaocheng University, Liaocheng 252059, China; YCN5053@163.com (C.-N.Y.); q893614459@163.com (Q.-D.L.); 17763557321@163.com (W.Z.); 17863525659@163.com (R.J.); 17863523877@163.com (C.-Z. L.); wangshuagnshuang@lcu.edu.cn (S.-S.Wang.)

[2] College of Materials Science and Engineering, Qingdao University, Qingdao 266071, China; xulinbang@163.com (Xu. L.)

* Correspondence: wangliping5@163.com(L.-P.W.); lglzsd@126.com (G.L.); Tel.: +86-635-8230-919 (L.-P.W.)

Received: 16 June 2019; Accepted: 09 July 2019; Published: 23 July 2019

Table. S1 Molecular weight and distribution of PS in solution

Sample	Mn [a]	Mw [a]	PDI [b]
SNPs-*g*-PS	6995	8428	1.20

Molecular weight of free PS obtained from the solution determined by GPC using narrow PS standards, THF as the eluent.
